# Psychosocial burden of healthcare professionals in times of COVID-19 – a survey conducted at the University Hospital Augsburg

**DOI:** 10.3205/000281

**Published:** 2020-06-22

**Authors:** Giulia Zerbini, Alanna Ebigbo, Philipp Reicherts, Miriam Kunz, Helmut Messman

**Affiliations:** 1 Department of Medical Psychology and Sociology, Medical Faculty, University of Augsburg, Germany; 2 Medical Clinic III, University Hospital Augsburg, Germany

**Keywords:** COVID-19, mental health, psychological burnout, health resources, health personnel

## Abstract

**Objective:** The outbreak of COVID-19 was declared a pandemic by the WHO in March 2020. Studies from China, where the virus first spread, have reported increased psychological strain in healthcare professionals. The aim of this study was to investigate the psychosocial burden of physicians and nurses depending on their degree of contact with COVID-19 patients. In addition, we explored which supportive resources they used and which supportive needs they experienced during the crisis.

**Methods:** Data were collected between March and April 2020 at the University Hospital Augsburg. A total of 75 nurses and 35 physicians, working either in a special COVID-19 ward or in a regular ward, took part in the survey. The participants filled in two standardized questionnaires (the Patient Health Questionnaire, PHQ; and the Maslach Burnout Inventory, MBI), and reported their fear of a COVID-19 infection and stress at work on a 10-point Likert scale. Finally, they answered three open-ended questions about causes of burden, supportive resources and needs during the crisis.

**Results:** Nurses working in the COVID-19 wards reported higher levels of stress, exhaustion, and depressive mood, as well as lower levels of work-related fulfilment compared to their colleagues in the regular wards. Physicians reported similar scores independent of their contact with COVID-19 patients. The most common causes for burden were job strain and uncertainty about the future. Psychosocial support as well as leisure time were listed as important resources, and a better infrastructure adjustment to COVID-19 at the hospital (e.g. sufficient staff, keeping teams and working schedules stable) as suggestion for improvement.

**Conclusions:** Our findings indicate that especially nurses working in COVID-19 wards are affected psychologically by the consequences of the pandemic. This might be due to a higher workload and longer time in direct contact with COVID-19 patients, compared to physicians.

## Zusammenfassung

**Ziel:** Im März 2020 wurde der Ausbruch von COVID-19 von der WHO zur Pandemie erklärt, nachdem sich der Virus zunächst vor allem in China ausbreitete. Erste Studien berichteten von einer erhöhten psychologischen Belastung der Beschäftigten im chinesischen Gesundheitssystem. Das Ziel der vorliegenden Studie war es, die besonderen psychosozialen Belastungen von Ärztinnen und Ärzten im Vergleich zu Pflegerinnen und Pflegern durch COVID-19 zu erfassen, unter Berücksichtigung des Ausmaßes der Exposition zu COVID-19-Patientinnen und -Patienten. Zusätzlich wollten wir besonders relevante Ressourcen und Möglichkeiten zur Entlastung eruieren.

**Methoden:** Die Datenerhebung erfolgte zwischen März und April 2020 am Universitätsklinikum Augsburg. Insgesamt nahmen 75 Pflegerinnen und Pfleger sowie 35 Ärztinnen und Ärzte an der Untersuchung teil, die entweder in regulären oder speziellen COVID-19-Stationen tätig waren. Neben zwei Standardinstrumenten (Gesundheitsfragebogen PHQ und Fragebogen zur Arbeitsbelastung MBI) bewerteten die Teilnehmenden ihre Angst, sich mit COVID-19 zu infizieren, und das Ausmaß der Belastung am Arbeitsplatz auf 10-stufigen numerischen Ratingskalen. Zusätzlich wurden drei offene Fragen zu den größten Belastungen, Ressourcen und Bedürfnissen durch bzw. in der Krise beantwortet.

**Ergebnisse:** Insbesondere Pflegerinnen und Pfleger auf COVID-19-Stationen berichteten mehr Stress, Ermüdung, depressive Symptome und geringere Erfüllung am Arbeitsplatz als ihre Kolleginnen und Kollegen auf den regulären Stationen. Ärztinnen und Ärzte hingegen erzielten unabhängig von der spezifischen Exposition zu COVID-19-Patientinnen und -Patienten ähnliche Ergebnisse. Am häufigsten wurden von den Teilnehmenden das Arbeitspensum und die Ungewissheit hinsichtlich der weiteren Entwicklung als Ursache ihrer Belastung benannt. Psychosoziale Unterstützung und Freizeit wurden als wichtige Ressourcen aufgelistet. Die Teilnehmenden mahnten u.a. an, auf ausreichendes Personal, Kontinuität der Teamzusammensetzung und Schichtplanung zu achten.

**Schlussfolgerungen:** Unsere Ergebnisse zeigen, dass insbesondere das Pflegepersonal – im Kontrast zu Ärztinnen und Ärzten – auf COVID-19-Stationen psychosozial unter den Folgen der Pandemie leidet, vermutlich als Konsequenz der Mehrarbeit und des höheren Expositionsrisikos.

## Introduction

The outbreak of COVID-19 in December 2019 in China quickly became a worldwide threat and was declared a pandemic by the World Health Organization on March 11^th^ 2020. The implications are enormous on many different levels, from health (physical and psychological) to socio-political and economical concerns.

Healthcare professionals occupy the front line during epidemics/pandemics and are at increased risk concerning their physical and mental health [1], [2], [3]. Reasons for decreased mental health comprise, among others, excessive workload, unpreparedness, and emotional distress (e.g. fear of infection, concerns about family) [2], [4]. Compared to previous epidemics, the COVID-19 pandemic might be more challenging because of some specific features of the virus, such as the high contagiousness, a rather low level of knowledge regarding the course of infection and (long-term) consequences, and a lack of established treatments or vaccines [5]. Moreover, for most hospitals worldwide this is an unprecedented scenario, which is accompanied by great challenges regarding various aspects of health care such as hygiene concepts, sufficient protective measures and equipment, expansion of intensive care units, etc.

In addition to the short-term psychological strain, factors such as quarantine, working in high-risk wards, and being in contact with infected patients were shown to be associated with long-term post-traumatic symptoms during the SARS (Severe Acute Respiratory Syndrome) epidemic [6]. It is therefore essential to carefully monitor healthcare professionals and to pinpoint possible sources and consequences of distress in order to improve their present condition, and even more so to limit long-term negative psychological effects.

The first studies (all cross-sectional) on the psychological burden of healthcare professionals in association with the COVID-19 pandemic have recently been published and were mainly conducted in China. Increased levels of anxiety, depression, and stress were reported as common psychological consequences, while social support, suitable protective equipment, clear guidelines, and job recognition were indicated as important resources [7], [8], [9], [10], [11]. Due to the worldwide spread of COVID-19, more studies exploring the psychosocial burden and individual sources of resilience in healthcare workers are needed in other countries as well.

The aim of this study was to explore whether individuals working in special COVID-19 wards are experiencing a higher psychosocial strain compared to their colleagues working in regular wards, and whether different healthcare professionals (nurses vs. physicians) are differently affected by the pandemic. In addition, we used open-ended questions to explore individual reasons and perceptions of psychosocial burden and possible solutions for an improvement of the working conditions.

## Methods

The study was evaluated and approved by the Institutional Review Board of the University Hospital Augsburg (BKF study no. 2020-14), and was conducted between March 23^rd^ and April 24^th^ 2020 at the University Hospital Augsburg, Germany. During the study period, a total of 272 patients were hospitalized in COVID-19 wards at the University Hospital Augsburg with a suspected diagnosis of COVID-19. After being tested for COVID-19, 83 were found positive. Out of these 83 patients, 30 had to be admitted to the COVID-19 intensive care unit. The 189 patients who tested negative left the COVID-19 wards after a couple of days and were transferred to regular wards (normal or intensive care wards).

### Participants and questionnaires

A total of 111 health professionals took part in the survey. One participant did not report their type of profession and gender, leaving a final sample size of N=110 (77 females). Health professionals either worked at “regular wards” (normal and intensive care wards as well as endoscopic ward without or with very little contact with COVID-19 patients), or at “COVID-19 wards” (normal and intensive care wards with COVID-19 patients).

75 nurses (45 COVID-19 wards vs. 30 regular wards) and 35 physicians (17 COVID-19 wards vs. 18 regular wards) filled in the Patient Health Questionnaire (PHQ) [12] and the German version of the Maslach Burnout Inventory (MBI) [13]. The PHQ is a widely used self-report diagnostic tool for mental health symptoms and comprises 5 subscales. For this study, we used the “depression”, “anxiety”, and “stress” subscales. The answer options for the first 2 subscales were given on a 4-point frequency Likert scale (0=not at all, 1=several days, 2=more than half the days, 3=nearly every day), with possible scores ranging between 0 and 27 for the “depression” subscale, and between 0 and 21 for the “anxiety” subscale. The “stress” subscale used a 3-point Likert scale (0=not affected, 1=a little affected, 2=very affected), with possible scores ranging between 0 and 18. The MBI was developed to specifically assess burnout symptoms of workers in the human/social services. The symptoms are divided into the subscales of “exhaustion”, “depersonalization”, and “fulfilment”. Answer options were given on a 6-point frequency Likert scale (0=never, 1=almost never, 2=sometimes, 3=often, 4=very often, 5=always), and possible score ranges were 0–45 for “exhaustion”, 0–25 for “depersonalization”, and 0–40 for “fulfilment”. Participants also rated how much they feared to be infected with COVID-19 and how stressed they felt at their job because of the current situation (pandemic) on a 10-point Likert scale. These two questions were developed to be COVID-19-specific. Finally, participants answered three open-ended questions about causes of burden, supportive resources, and suggestions to improve the current working conditions.

### Statistical analysis

Statistical analysis and graphs were performed with R software (version 4.0.0).

Across all groups: We first correlated (using Spearman’s rank correlation) the COVID-19-specific questions (fear to be infected and stress at work) with each PHQ and MBI subscale to explore whether there was a concordance between COVID-19-specific and general measures of psychological burden.

Between groups: We then compared questionnaire scores, fear of infection, and stress at work ratings using ANOVAs with a 2x2 design, employing profession (physicians vs. nurses) and ward (COVID-19 vs. regular) as between-subject factors. Normality distribution of residuals (qq-plots) and homogeneity of variance across groups were tested and the assumptions for ANOVA were met. When the interaction effect was significant (p<0.05), post-hoc tests were performed using independent-sample t-tests.

Open-ended answers were analyzed by quantitative content analysis with data-driven category development. First, two experts developed the categories independently. Then, the categories were revised and descriptors for each category were defined during a panel discussion with two additional experts. Each open-ended answer was categorized, and percentages were calculated (number of participants naming a specific category for one open-ended question divided by the total number of participants answering that open-ended question multiplied by 100). Table 1 (Tab. 1) summarizes the categories and descriptors developed for the three open-ended questions. When a category was reported by more than 25% of the participants, we compared which percentage of nurses vs. physicians and of participants working in regular vs. COVID-19 wards chose that category.

## Results

### Questionnaires

**Figure 1:**
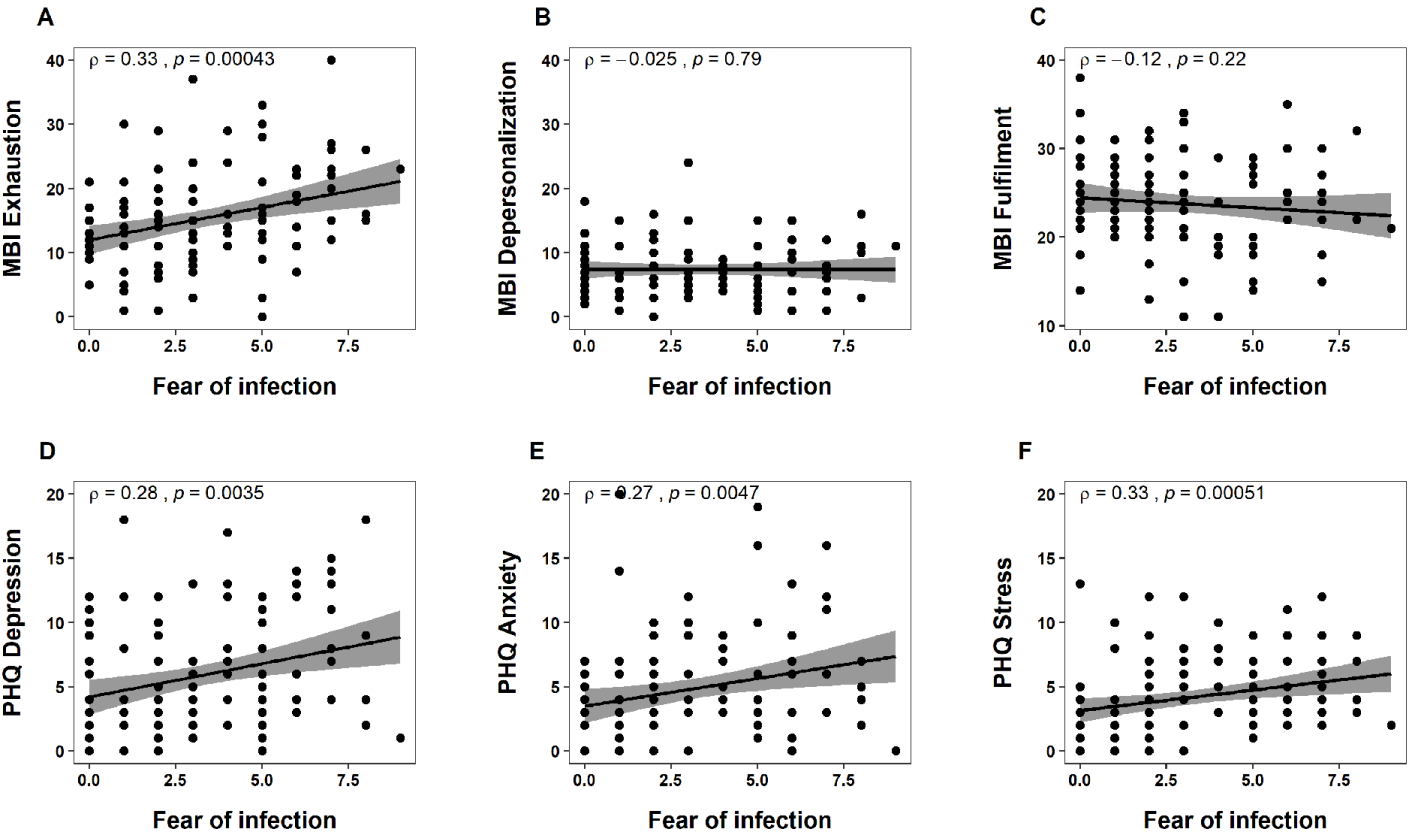
Spearman correlations between the rating scale fear of infection (x-axes) and MBI subscales A) exhaustion, B) depersonalization, C) fulfilment, and PHQ subscales D) depression, E) anxiety, F) stress (y-axes) are shown. The grey area around the regression line indicates the 95% confidence interval. On the top left of each panel the rho (ρ) statistic and p-value (p) of the correlations are reported. Fear of infection was positively associated with exhaustion, depression, anxiety, and stress scores (p<0.05).

Across all groups: The fear of being infected with COVID-19 positively correlated with the MBI exhaustion subscale (ρ=0.33, p=0.0004) and with the three PHQ subscales (depression: ρ=0.28, p=0.0035; anxiety: ρ=0.27, p=0.0047; stress: ρ=0.33, p=0.0005). Participants with increased scores for exhaustion, depression, anxiety, and stress reported a higher fear of being infected with the virus (Figure 1 (Fig. 1)).

**Figure 2:**
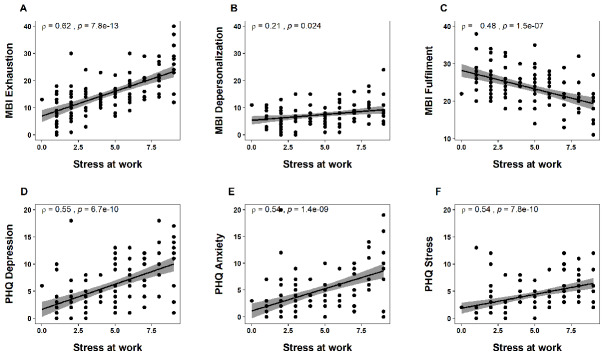
Spearman correlations between the rating scale stress at work (x-axes) and MBI subscales A) exhaustion, B) depersonalization, C) fulfilment, and PHQ subscales D) depression, E) anxiety, F) stress (y-axes) are shown. The grey area around the regression line indicates the 95% confidence interval. On the top left of each panel the rho (ρ) statistic and p-value (p) of the correlations are reported. Stress at work was significantly associated with all questionnaire scores (p<0.05).

Similarly, participants who reported to currently feel more stressed at work showed increased burnout symptoms and psychological strain (Figure 2 (Fig. 2)). Here, the strength of the correlations was higher compared to the fear rating scale (exhaustion: ρ=0.62, p<0.0001; depersonalization: ρ=0.21, p=0.0242; fulfilment: ρ=–0.48, p<0.0001; depression: ρ=0.55, p<0.0001; anxiety: ρ=0.54, p<0.0001; stress: ρ=0.54, p<0.0001).

**Figure 3:**
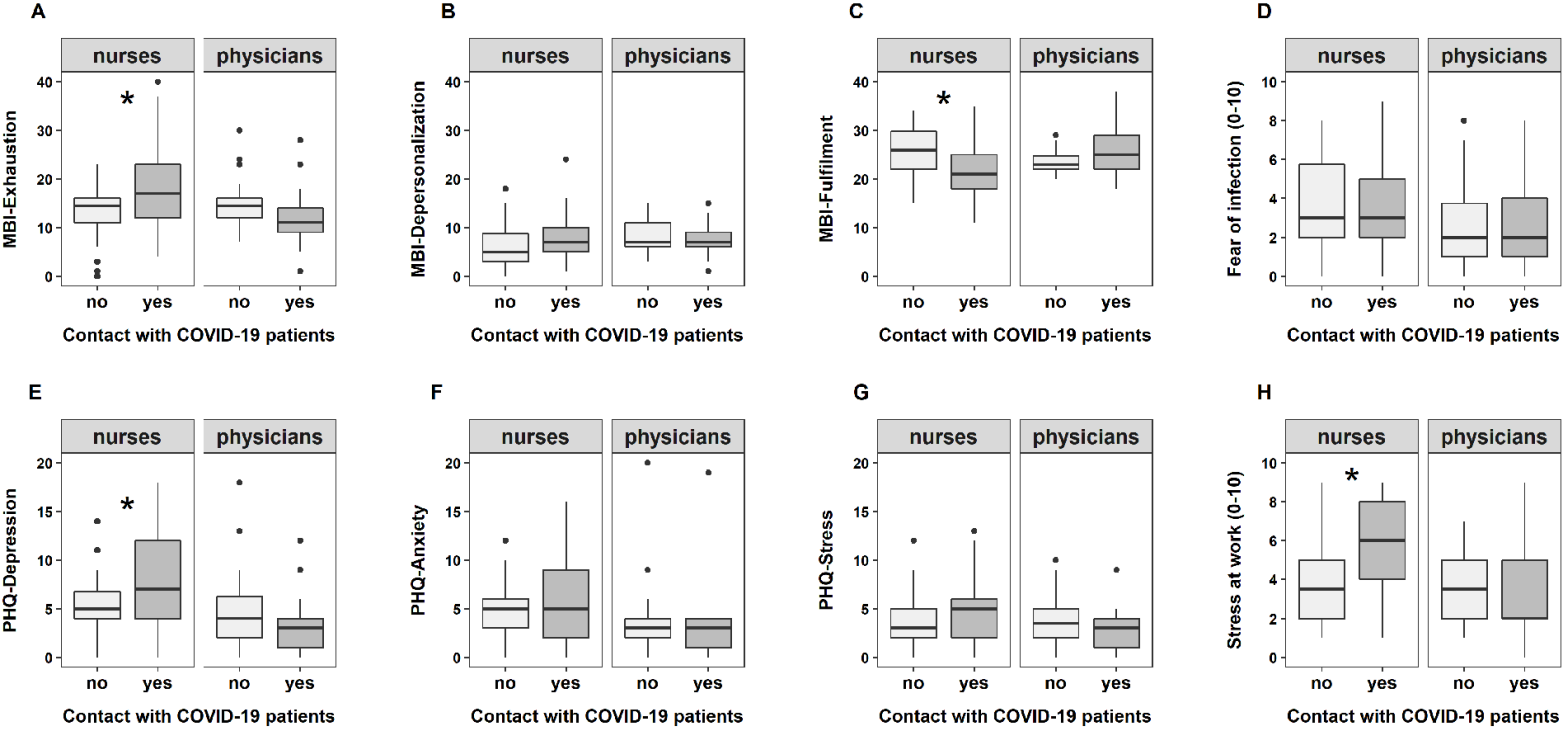
Questionnaire and rating scale scores were compared between COVID-19 wards and regular wards, for nurses and physicians separately. Nurses working in direct contact with COVID-19 patients reported significantly higher exhaustion (A), depressive mood (E), stress at work (H), and lower fulfilment (C). Boxplots show the median and the interquartile range (25^th^ to the 50^th^ percentiles) of the raw data. The whiskers stretch out to the smallest and the largest values within 1.5 times from the interquartile range. Dots indicate values outside these limits. P-values (*p<0.05) reflect the p-values calculated with independent sample t-tests (post hoc analyses).

Between groups: Comparing questionnaire scores as well as fear of infection and stress at work ratings between groups revealed no significant main effects for profession or ward (all p-values >0.05) (Figure 3 (Fig. 3)). However, we found significant interactions between these factors for the PHQ subscale depression (F_1,106_=5.380, p=0.0223), the MBI subscales exhaustion (F_1,106_=7.874, p=0.0060) and fulfilment (F_1,106_=10.014, p=0.0020), and the stress at work rating scale (F_1,106_=5.082, p=0.0262). Nurses working in the COVID-19 wards reported higher levels of depressive mood (t(73)=–3.066, p=0.0030), exhaustion (t(73)=–2.970, p=0.0040), and lower levels of fulfilment (t(73)=3.246, p=0.0018) compared to colleagues working in the regular wards. Physicians, on the contrary, reported similar scores irrespective of the type of ward (all p-values >0.05). Similarly, stress at work ratings were significantly higher for nurses working in the COVID-19 wards compared to regular wards (t(73)=–3.245, p=0.0018), whereas this difference could not be found in physicians (p>0.05).

### Open-ended questions

**Figure 4:**
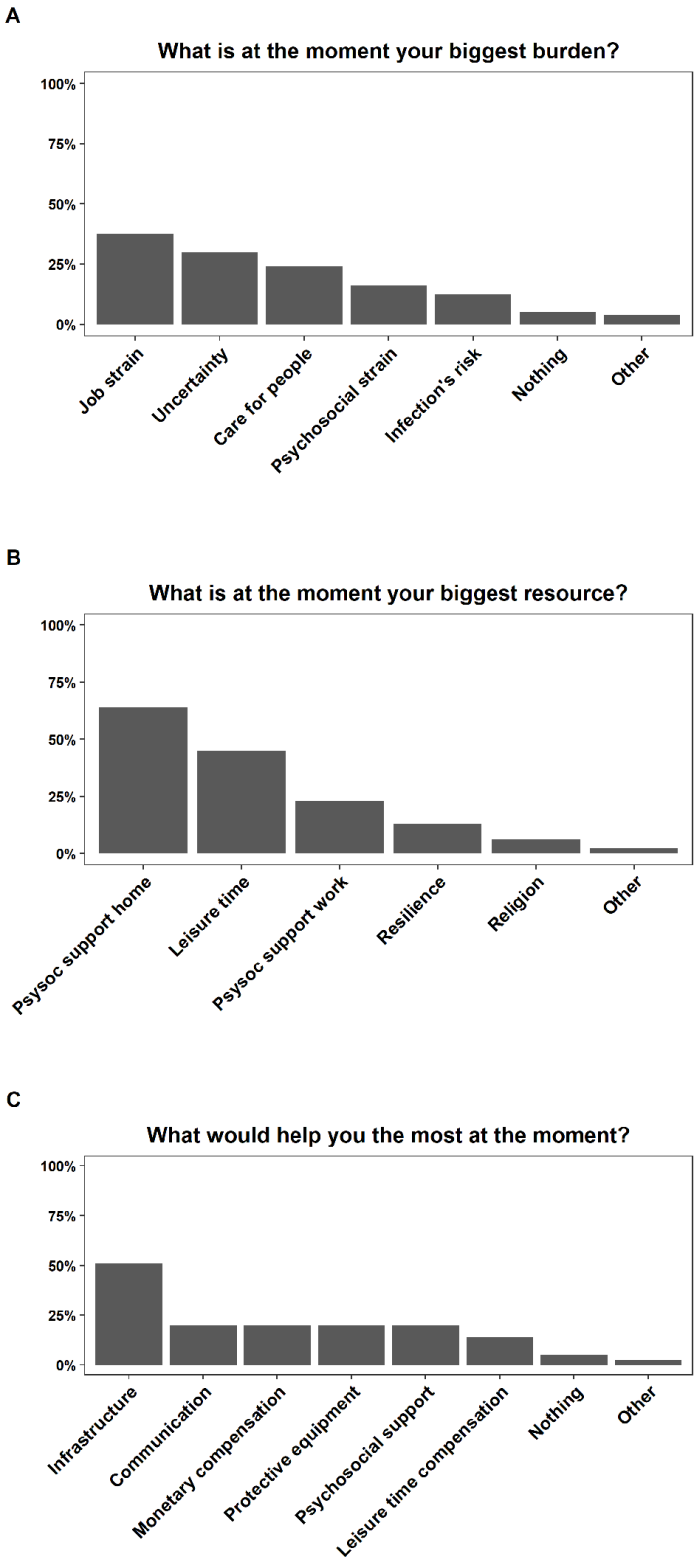
An overview of the categories and their frequencies developed for each open-ended question is shown. Panel A: Participants reported job strain (37.5%) and uncertainty (30%) as the most common causes for their burden, followed by care for people (23.8%), psychosocial strain (16.3%), and risk of infection (12.5%). Panel B: Participants reported psychosocial support by family and friends (64.3%) as well as leisure time (45.3%) as important resources, followed by psychosocial support at work (22.6%), personal resilience factors (13.1%), and religion (6%). Panel C: 51% of the participants wished for a better infrastructure adjustment to COVID-19 at the hospital. Other suggestions for improvement comprised better communication (20.3%), more monetary compensation (20.3%), more leisure time compensation (13.9%), adequate protective equipment (20.3%), and better psychosocial support (20.3%).

Figure 4 (Fig. 4) shows an overview of the categories and their frequencies developed for each open-ended question. Participants reported job strain (37.5%) and uncertainty (30%) as the most common causes for their burden, followed by care for people (23.8%), psychosocial strain (16.3%), and risk of infection (12.5%). The two categories that were reported more frequently (cutoff: 25%) were then further investigated considering profession and ward. 55% of the nurses in the COVID-19 ward against only 12.5% in the regular ward complained about job strain. For the physicians, the results revealed an opposite pattern, with 25% reporting job strain as a burden in the COVID-19 ward against 54% in the regular ward. Uncertainty about the future (economic, health) was reported by 50% of the nurses and 31% of the physicians as a burden in the regular ward, compared to 22.5% of the nurses and 8% of the physicians in the COVID-19 ward.

Most of the participants reported psychosocial support by family and friends (64.3%) as well as leisure time (45.3%) as important resources, followed by psychosocial support at work (22.6%), personal resilience factors (13.1%), and religion (6%). Psychosocial support by family and friends as well as leisure time were similarly important for nurses and physicians in both wards (COVID-19 and regular). The only clear difference was between physicians in the two wards, with almost all the physicians (92%) in the COVID-19 ward reporting family and friends as resource, compared to 58% in the regular ward.

The most common suggestion for improvement referred to a better infrastructure adjustment to COVID-19 at the hospital: 51% of the participants answering the open-ended questions wished for more staff and/or rooms and space, better organization and planning (e.g. staff roster, standardized procedure with COVID-19 patients), and that people working together in a team should be kept stable. The suggestion for better infrastructure came mainly from people working at COVID-19 wards (62% of the nurses and 85% of the physicians). Other suggestions for improvement comprised better communication (20.3%), more monetary compensation (20.3%), more leisure time compensation (13.9%), adequate protective equipment (20.3%), and better psychosocial support (20.3%).

## Discussion

Epidemics have always been part of human history. However, a pandemic of such dimension as the COVID-19 one is rare. For the first time in recent history, almost the entire world was in lockdown. The mental and physical health of healthcare workers in particular are at risk during epidemics [1], [2], [3]. Here, we investigated the psychosocial burden of healthcare professionals working in direct contact with COVID-19 patients (compared to colleagues working in regular wards) and explored whether nurses and physicians are differently affected by the pandemic.

Overall, specific indicators of COVID-19-related consequences (i.e. fear of infection and stress at work) were associated with more general measures of psychological strain and burnout (PHQ and MBI scores), indicating that the COVID-19 pandemic is an emotionally and physically stressful event. However, our findings indicate that not all healthcare professionals are affected equally by the COVID-19 pandemic. It was especially the group of nurses working in COVID-19 wards who was affected psychologically by the consequences of the pandemic. They reported higher exhaustion, depressive symptoms, and lower job fulfilment together with an overall higher stress perception at work compared to their colleagues in regular wards. Physicians working in COVID-19 wards did not report increased psychological burden. This difference between nurses and physicians might be due to a higher workload and longer time in direct contact with COVID-19 patients experienced especially by the nurses.

Our results are in line with the study of Lai et al. [8] who found that nurses and healthcare workers in direct contact with COVID-19 patients were among the participants reporting the highest psychological burden. Similarly, participants suffering more from anxiety and general mental health disturbances were found to be either nurses [9] or the ones with higher contact with COVID-19 patients [7].

The open-ended questions revealed that the most common causes for psychosocial burden were job strain (increased workload, organizational changes in working team, conflicts with colleagues) and uncertainty about the future (healthcare system and economic crisis). Concerns about one’s safety and the safety of the family, as well as the mortality reports from COVID-19 infection, have also been reported as afflicting factors [9]. Similar to other studies, we found that social support was one of the most important resources to cope with the psychological burden following the pandemic [9], [11]. Finally, our participants expressed the need for a better infrastructure adjustment to the crisis. This included, among others, sufficient staff members to face the increased number of patients, clear organization and planning (e.g. composition of working teams, scheduling of working hours). Similar needs were expressed in another recent study, together with the demand for more information about the pandemic, clearer guidelines and improved communication from the heads of the hospital/units, which were also mentioned in our study [9].

When interpreting the results of the current study, one needs to consider that data were collected in a single hospital in Germany and generalization of our findings might be limited to similar circumstances. Moreover, our relatively small sample size did not allow more in-depth analyses (such as comparing regular and intensive wards). More studies are needed to replicate our findings in other hospitals and countries and to extend our results, for example by comparing different types of wards (with more or less contact with COVID-19 patients) and different types of healthcare professionals (not only nurses and physicians).

## Conclusion

Altogether, our study demonstrates that healthcare workers, especially the ones in constant and direct contact with COVID-19 patients, are at higher risk for psychological burden. Given that nurses seem to be more affected, special programs addressing their needs are required. Based on the current findings that family and friends as well as leisure time were reported as resilience factors by most of the participants, off-work time should be valued and not jeopardized by long working hours. Psychosocial support at work appeared to be another important resource. Keeping working teams stable, improving communication and recognition, providing clear guidelines and social support are examples of how the working environment could be improved during epidemics [14].

## Notes

### Competing interests

The authors declare that they have no competing interests.

### Authors’ contributions

Giulia Zerbini and Alanna Ebigbo contributed equally to this work.

### Acknowledgment

We thank Franziska Moll for her help in organizing and digitalizing the data.

### Availability of data and materials

The data analyzed in this study is available from the corresponding author on request.

## Figures and Tables

**Table 1:**
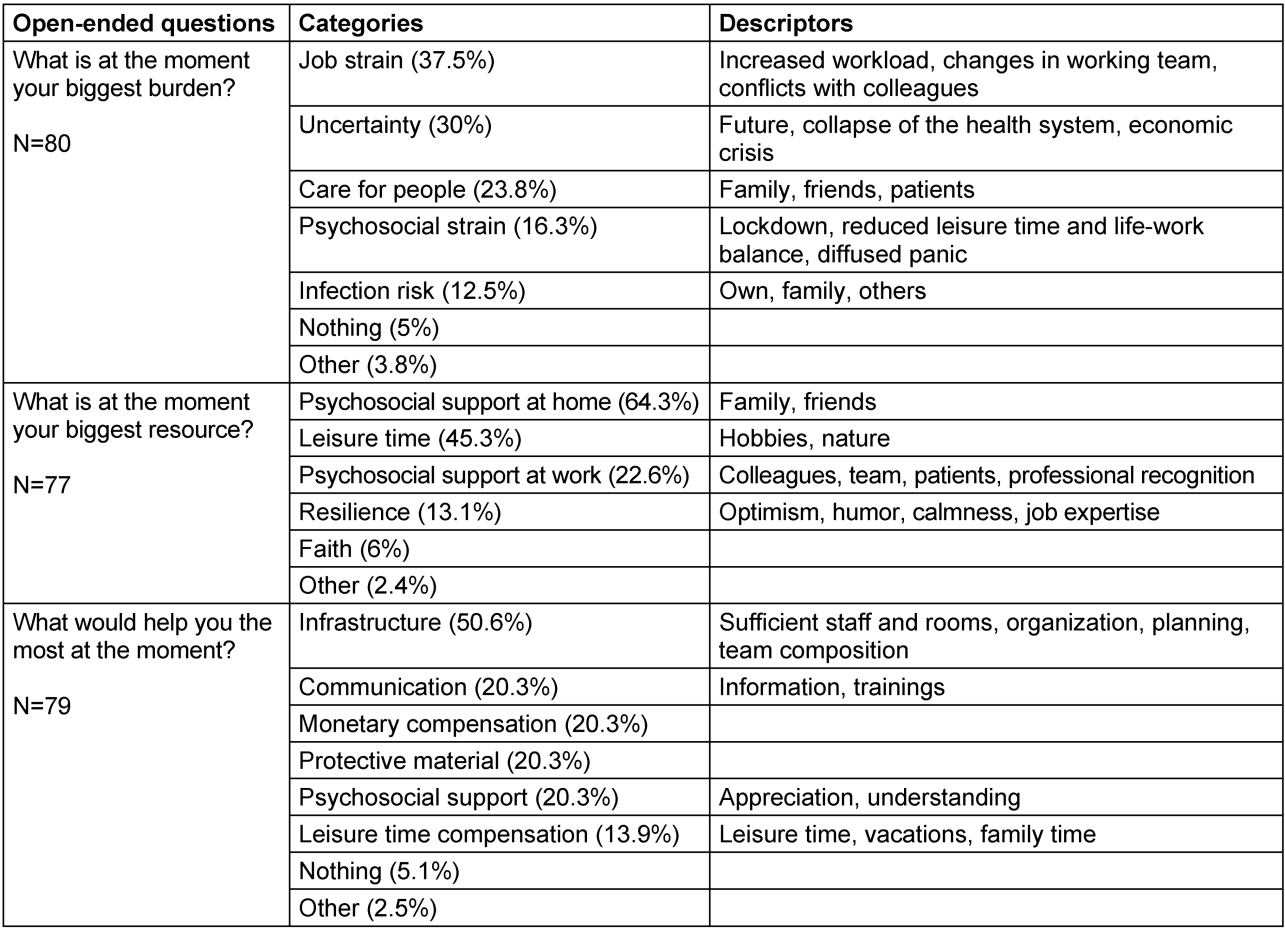
Categories and descriptors developed for the three open-ended questions. The number of participants answering each open question is also reported.
